# Analysis of Frequency Response and Scale-Factor of Tuning Fork Micro-Gyroscope Operating at Atmospheric Pressure

**DOI:** 10.3390/s150202453

**Published:** 2015-01-22

**Authors:** Xukai Ding, Hongsheng Li, Yunfang Ni, Pengcheng Sang

**Affiliations:** 1 School of Instrument Science and Engineering, Southeast University, Nanjing 210096, China; E-Mails: dingxukai@126.com (X.D.); niyunfang@126.com (Y.N.); 15805153292@163.com (P.S.); 2 Key Laboratory of Micro-Inertial Instrument and Advanced Navigation Technology of Ministry of Education, Southeast University, Nanjing 210096, China

**Keywords:** MEMS gyroscope, frequency response, scale-factor, atmospheric pressure, interference mode, demodulation phase

## Abstract

This paper presents a study of the frequency response and the scale-factor of a tuning fork micro-gyroscope operating at atmospheric pressure in the presence of an interference sense mode by utilizing the approximate transfer function. The optimal demodulation phase (ODP), which is always ignored in vacuum packaged micro-gyroscopes but quite important in gyroscopes operating at atmospheric pressure, is obtained through the transfer function of the sense mode, including the primary mode and the interference mode. The approximate transfer function of the micro-gyroscope is deduced in consideration of the interference mode and the ODP. Then, the equation describing the scale-factor of the gyroscope is also obtained. The impacts of the interference mode and *Q*-factor on the frequency response and the scale-factor of the gyroscope are analyzed through numerical simulations. The relationship between the scale-factor and the demodulation phase is also illustrated and gives an effective way to find out the ODP in practice. The simulation results predicted by the transfer functions are in close agreement with the results of the experiments. The analyses and simulations can provide constructive guidance on bandwidth and sensitivity designs of the micro-gyroscopes operating at atmospheric pressure.

## 1. Introduction

In recent years, MEMS inertial devices have been widely adopted for many types of consumer electronic products, including phones, tablets, gaming system, toys and emerging wearable gadgets [1]. The micro-gyroscopes used in these consumer electronics are generally classified as rate-grade devices [2]. Although micro-gyroscopes have many advantages over traditional gyroscopes for their small size, low power consumption, low cost and batch fabrication, high performance micro-gyroscopes are still too expensive for consumer products, even for industrial products.

Packaging, as one of the key manufacturing processes of MEMS sensors, provides protection from the environment, such as mechanical protection, optical and thermal protection and electrical interface and isolation. The packaging cost of MEMS devices in general is up to 70% of total costs [3]. The requirements for packaging of MEMS sensors are generally different among sensor types. Often, vacuum packaging is required for micro-gyroscopes to avoid viscous damping. Steps to attain and maintain the vacuum ambient include providing a means to pump away gases that filled with the package, hermetically sealing the package and proper process to reduce trapped gases in the package [4]. Proper packaging processes and getter technology are vital to success in vacuum packaging, but all of these processes increase the cost of micro-gyroscopes. Developing high-performance micro-gyroscopes operating at atmospheric pressure is an effective means to reduce the cost. A micro-gyroscope working at atmospheric pressure, different from a vacuum packaged one, has a low *Q*-factor and possibly large coupling damping due to the viscous air surrounding the movable structures.

In 2002, researchers in Analog Devices reported a single-chip, surface micro-machined integrated gyroscope with atmospheric hermetic package [5]. This micro-gyroscope achieved a Root Allan variance of 50°/h with a full scale range of ±150°/s. In 2007, a silicon-on-insulator MEMS gyroscope operating at atmospheric pressure with a short-term bias stability of 1.5°/s was reported in [6]. During 2008–2010, researchers at Peking University developed a series of micro-gyroscopes working at atmospheric pressure [7–13]. In [7,8], the authors described micro-gyroscopes operating at atmospheric pressure with small cross-talks between drive mode and sense mode. In [9–13], the authors reported several lateral-axis micro-gyroscopes which could work at atmospheric pressure. They developed novel torsional sensing comb capacitors to lower the air damping and electrostatic force balanced combs to suppress the mechanical coupling.

Some reported researches improved the *Q*-factor of the gyroscopes operating under air pressure through special structure design. Slide film damping effects in drive and sense modes were used to achieve large quality factors of gyroscopes even operating at atmospheric pressure [14]. The sensitivity of a slot-structure micro-gyroscope working at atmosphere was improved through a tunable electrostatic spring constant which was achieved by triangular shape fixed electrodes [15]. Some researchers discussed the coupling error in the vibratory MEMS gyroscope with various air damping in detail [16].

The frequency response and the scale-factor of the gyroscopes have not been paid close attention in the literatures mentioned above. The frequency responses of gyroscopes with high *Q*-factor have been well studied [17,18]. However, as for gyroscopes operating at atmospheric pressure, the low *Q*-factor has a great impact on the characteristics of the frequency response and scale-factor. In [17], the demodulation phase, which is negligible for high *Q* cases but significant for low *Q* cases, is not included in the theoretical analysis. In [19], collateral modes of micro-gyroscopes, which provide mechanisms for the transfers of energy that are independent of angular rate, were analyzed through a three DOF model. The effects of the additional modes on the bias of micro-gyroscopes were studied specifically.

The work reported in this paper focuses on the frequency response and scale-factor of the gyroscope with low *Q*-factor. Not only the demodulation phase but also an observed interference sense mode is taken into consideration in the analysis. This paper is organized as follows. Section 2 introduces the investigated micro-gyroscope with its imperfections in the practical implementation. The transfer function of the sense mode consisting of the primary and the interference modes is given in Section 3. The equation of the optimal demodulation phase (ODP) is also presented. In Section 4, the approximate transfer function of the gyroscope is deduced, followed by a series of simulations and analyses centered on the frequency response. Section 5 shows the simulations and analyses of the scale-factor. The results of the experiments, which verify the theoretical analyses carried out in the former sections, are shown in Section 6. At last, Section 7 concludes this paper.

## 2. Imperfections of the Tuning Fork Micro-Gyroscope Operating at Atmospheric Pressure

**Figure 1.**
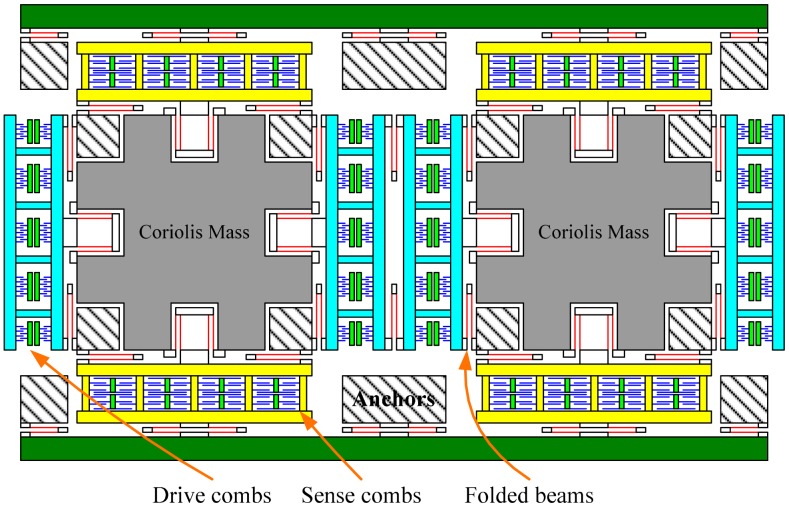
Simplified schematic of the tuning fork micro-gyroscope with mechanically coupled drive mode and sense mode.

The tuning fork micro-gyroscope operating at atmospheric pressure has large proof masses to increase the signal noise ratio of the Coriolis response and has a large drive force to increase the vibration amplitude in the viscous air. The simplified schematic of the tuning fork micro-gyroscope with mechanically coupled drive mode and sense mode is shown in Figure 1 [18]. The sense combs are configured as squeeze mode to improve the sensitivity. The proof mass, or the Coriolis mass, is the central mass in the structure which transfers the vibration energy from the drive mode to the sense mode through Coriolis Effect. As illustrated in Figure 1, the micro-gyroscope has two identical proof masses whose working vibration directions are always opposite. This is why this kind of micro-gyroscope is named as tuning fork gyroscope. This configuration produces a differential signal and rejects acceleration from the environment, which is common-mode to the structure.

**Figure 2.**
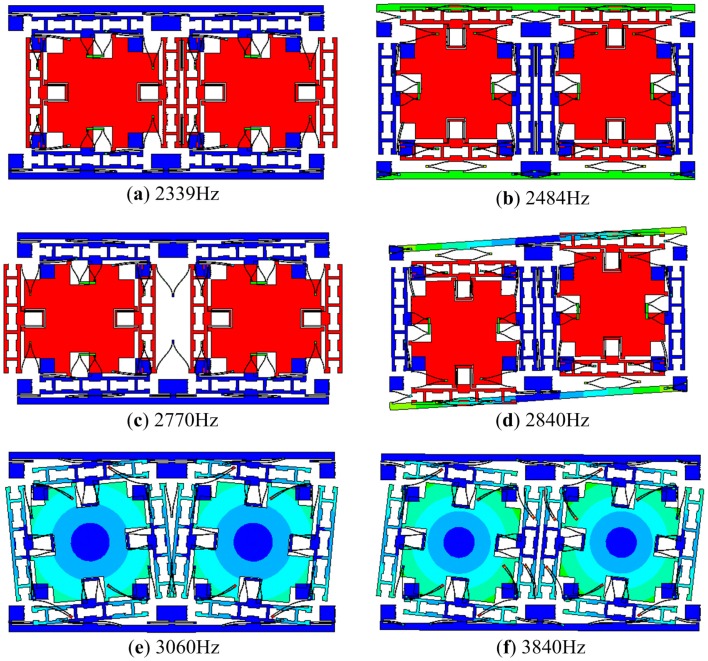
The first six modes of the micro-gyroscope: (**a**) in-phase mode in drive direction; (**b**) in-phase mode in sense direction; (**c**) anti-phase mode in drive direction; (**d**) anti-phase mode in sense direction; (**e**) anti-phase torsional mode; (**f**) in-phase torsional mode.

The first six modes of the presented micro-gyroscope are demonstrated in Figure 2. The designed natural frequencies of these modes are indicated in the figure. The anti-phase modes in drive and sense directions are the working modes, or the primary modes, while the other modes are interference modes. If the structure of micro-gyroscope is perfectly symmetric and is loaded differentially, there will be only primary modes participating in the vibration.

### 2.1. Coupling Damping and Coupling Stiffness between the Drive Mode and the Sense Mode

Considering the primary modes and ignoring the nonlinearity which derives from the large deformation of the folded beams, the dynamic equation of the multi-DOF vibration system presented in Figure 1 can be modeled as

(1) $$\left[\begin{array}{cc} m_{x} & 0\\ 0 & m_{y} \end{array}\right]\;\left[\begin{array}{c} \ddot{x}\\ \ddot{y} \end{array}\right]+\left[\begin{array}{cc} c_{x} & c_{xy}\\ c_{yx} & c_{y} \end{array}\right]\;\left[\begin{array}{c} \dot{x}\\ \dot{y} \end{array}\right]+\left[\begin{array}{cc} k_{x} & k_{xy}\\ k_{yx} & k_{y} \end{array}\right]\;\left[\begin{array}{c} x\\ y \end{array}\right]=\left[\begin{array}{c} f_{x}\\ -2m_{c}\Omega \dot{x} \end{array}\right]$$

where *m_x_*, *m_y_* and *m_c_* represent the effective masses of the drive mode, the sense mode and the Coriolis mass, respectively; *x* and *y* represent the displacements of the drive mode and the sense mode; *c_x_* and *c_y_* are damping of each mode while *c_xy_* and *c_yx_* are coupling damping; *k_x_* and *k_y_* are stiffness of the modes while *k_xy_* and *k_yx_* are coupling stiffness; *f_x_* is the drive force applied to drive mode; *Ω* is the angular rate input to the sensor.

In the sense mode, due to the coupling damping and the coupling stiffness, there exists a quasi-stable vibration even in the absence of angular rate input as long as the drive mode is stably driven. The response of the spring force introduced by the coupling stiffness, widely known as quadrature error, is always orthogonal to the Coriolis response. The response of the viscous force introduced by the coupling damping is in-phase with the Coriolis response. These two kinds of couplings are the main sources of zero-rate output (ZRO) of the micro-gyroscope [20]. At the atmospheric pressure, the response of coupling damping is obvious, although not dominate compared with that of quadrature error. Generally, the ZRO caused by quadrature error can be eliminated by phase demodulation while the ZRO caused by coupling damping cannot be easily canceled out.

### 2.2. The Interference Modes in the Sense Mode

Actually, far more complicated than Equation (1) describes, besides the primary modes, the micro-gyroscope suffers from several interference modes introduced by mechanical coupling and multi-DOF in space. Even when the structure of the micro-gyroscope is forced differentially, there exist interference modes due to the asymmetries of the proof masses, the folded beams and the comb fingers. As long as the interference modes become pronounced because of severe asymmetries and can be observed in the sense direction, they will change the characteristics of the sense mode. A detailed analysis of in-phase mode in sense direction is reported in [18].

Moreover, once the interference modes appear, besides the angular rate applied to the sensitive axis of the micro-gyroscope, other motions, such as linear acceleration, vibration and rotation with respect to other axes, will cause responses in the sense direction. Linear acceleration or vibration along the sense direction will excite the in-phase mode in the sense direction. Under ideal conditions, this motion will not be detected thanks to the symmetric structure and the differential detection. However, under non-ideal conditions, this in-phase interference mode may be obviously observed in the sense direction. Hence, linear acceleration or vibration may cause a detectable response. Similarly, the out of plane modes, although not shown in Figure 2, will result in couplings from rotations applied to the non-sensitive axes.

In vacuum packaged gyroscopes, benefiting from the extremely low damping, the impacts of the interference modes on the primary mode are very small. However, considering the viscous air at the atmospheric pressure and the sense combs in squeeze mode, the *Q*-factor is expected to be very low. Thus, at least one observed interference mode which is located nearest to the primary mode needs to be taken into account when the frequency response of the sense mode is evaluated. The details will be analyzed in the next section.

## 3. Frequency Response of the Sense Mode and Optimal Demodulation Phase

Motivated by the mode superposition method which decomposes a linear multi-DOF vibration into a sum of several independent modes, the forced response of the sense mode can be viewed as the sum of the primary mode response and the interference mode response. The transfer function of the sense mode from applied force to displacement can be written as:

(2) $$G_{s}\left(s\right)=\frac{1}{m_{y}}\cdot \frac{1}{s^{2}+(\omega_{ny1}/Q_{1})s+\omega_{ny1}^{2}}+\frac{\alpha }{m_{y}}\cdot \frac{1}{s^{2}+(\omega_{ny2}/Q_{2})s+\omega_{ny2}^{2}}$$

(3) $$=\frac{1}{m_{y}}\frac{\left(1+\alpha \right)s^{2}+\left(\omega_{ny2}/Q_{2}+\alpha \omega_{ny1}/Q_{1}\right)s+\omega_{ny2}^{2}+\alpha \omega_{ny1}^{2}}{\left[s^{2}+(\omega_{ny1}/Q_{1}\right)s+\omega_{ny1}^{2}]\cdot [s^{2}+(\omega_{ny2}/Q_{2})s+\omega_{ny2}^{2}]}$$

where *ω_ny_*_1_ and *Q*_1_ are the natural frequency and the *Q*-factor of the primary mode, respectively; *ω_ny_*_2_ and *Q*_2_ are the natural frequency and the *Q*-factor of the interference mode, respectively; *α* is a constant coefficient related to asymmetries in the structure. The first term in Equation (2) represents the transfer function of the primary mode and the second term represents that of the interference mode. Since the two modes are independent, the sum of the two terms can describe the frequency response of the sense mode. The coefficient *α* is a weight of the interference mode and depicts the ratio of the peak amplitude of the interference mode to that of the primary mode.

Compared with a general sense mode described as a second-order system which has a pair of poles in the *s*-plane, this fourth-order system has two pairs of poles and a pair of zeros. The additional pairs of zeros/poles will distort the frequency response of the primary mode, especially the phase response which is of most concern.

**Figure 3.**
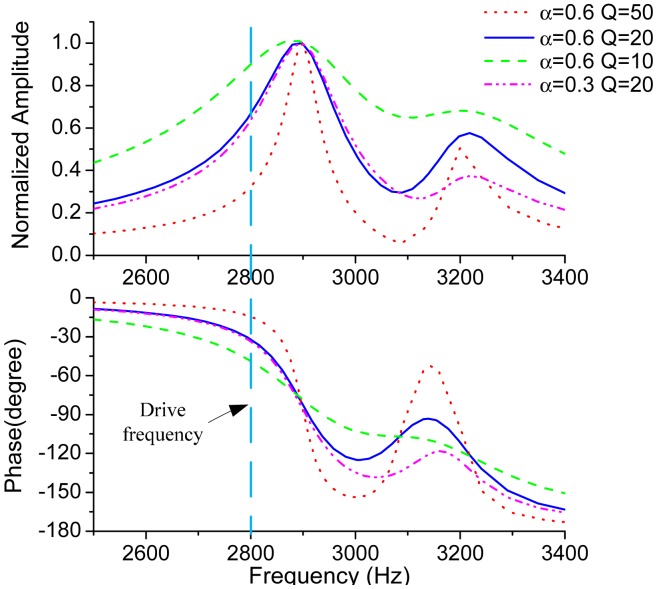
Frequency responses of the sense mode with different *Q*-factor and *α*.

Figure 3 plots the frequency responses of the sense mode by using Equation (2) with the parameters of *f_ny_*_1_ = 2900 Hz, *f_ny_*_2_ = 3200 Hz,*Q*_1_ = *Q*_2_ = *Q*. The parameter values used in the simulations are close to those of the tested micro-gyroscope. For the sake of clarity, the amplitudes of the primary peaks are normalized to 1. It can be concluded from the frequency responses that the amplitude and phase get steeper as *Q*-factor gets larger. The amplitude of the secondary peak is also determined by the coefficient *α*.

An assumed drive frequency of 2800 Hz is indicated in Figure 3. From the plot of phase response, the phase delay of the Coriolis response to the Coriolis force can be tens of degrees in low *Q*-factor cases. Therefore, in order to completely extract the Coriolis response, the reference signal used for phase demodulation, which is generally in-phase with the Coriolis force, should be delayed for an extra phase. This phase is called the ODP and denoted as *φ_od_*. From a physical viewpoint, *φ_od_* will be exactly the same with the phase delay of the Coriolis signal through the sense mode. By utilizing Equation (3), *φ_od_* can be given by

(4) $$\begin{array}{l} \varphi_{od}=-\text{arctan}\left(\frac{\omega_{d}\omega_{ny1}/Q_{1}}{\omega_{ny1}^{2}-\omega_{d}^{2}}\right)-\text{arctan}\left(\frac{\omega_{d}\omega_{ny2}/Q_{2}}{\omega_{ny2}^{2}-\omega_{d}^{2}}\right)\\ +\text{arctan}\left[\frac{\omega_{d}(\omega_{ny2}/Q_{2}+\alpha \omega_{ny1}/Q_{1})}{\omega_{ny2}^{2}+\alpha \omega_{ny1}^{2}-\left(1+\alpha \right)\omega_{d}^{2}}\right] \end{array}$$

where *ω_d_* is the working frequency of the drive mode. The first term in Equation (4) is determined by the primary mode while the last two terms reflect the phase impact of the nearest interference mode.

**Figure 4.**
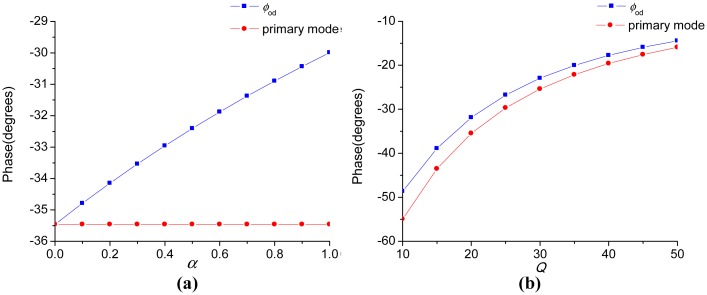
(**a**) The relationship between phase and *α* with the other parameters as *f_ny_*_1_ = 2900 Hz, *f_ny_*_2_ = 3200 Hz, *f_d_* = 2800 Hz and *Q*_1_=*Q*_2_ = 20;(**b**) The relationship between phase and *Q* with the other parameters as *f_ny_*_1_ = 2900 Hz, *f_ny_*_2_ = 3200 Hz, *f_d_* = 2800 Hz, *α* =0.6 and *Q*_1_=*Q*_2_ = *Q*.

Figure 4 exhibits the relationships between the ODP, the *Q*-factor and the coefficient α. The red curve denoted as “primary mode” in Figure 4 is the phase delay introduced by the primary mode only. As illustrated in Figure 4a, the ODP is dominantly determined by the primary mode and appreciably decreased by the interference mode. In Figure 4b, as *Q* becomes smaller, the ODP gets larger, as well as the impact of the interference mode. In fact, although not shown here, the ODP gets within −1° when *Q* reaches hundreds to thousands, which is the general case in vacuum packaged micro-gyroscopes. Thus, the demodulation phase is always set as 0° in the high-*Q* micro-gyroscopes for convenience. However, the phase delay in the micro-gyroscopes operating at atmospheric pressure is significant and must be dealt with carefully.

## 4. Frequency Response of the Micro-Gyroscope

### 4.1. The Approximate Transfer Function from the Angular Rate Input to the Output of the Micro-Gyroscope

In this subsection, the approximate transfer function of the micro-gyroscope from the angular rate input to the sensor output is deduced in consideration of the ODP and the interference mode. Assume that the displacement of the proof mass is

(5) $$x\left(t\right)=A_{x}\sin\left(\omega_{d}t\right)$$

where *A_x_* is the amplitude of the displacement. Substituting Equation (5) into Equation (1) gives the Coriolis force applied to the sense mode

(6) $$f_{c}\left(t\right)=-2m_{c}A_{x}\omega_{d}\cos\left(\omega_{d}t\right)\times \Omega \left(t\right)$$

**Figure 5.**
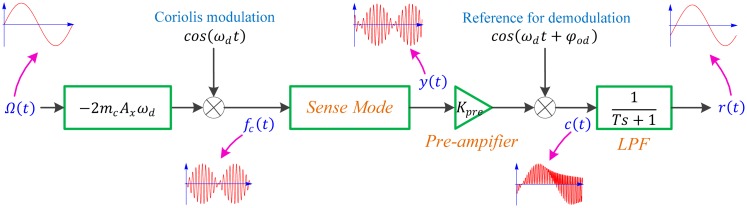
The sense block diagram of the micro-gyroscope.

The sense block diagram is presented in Figure 5 where *K_pre_* is the gain of the pre-amplifier which transfers the displacement of the structure into voltage and LPF is the low pass filter which attenuates the second harmonic produced by the demodulation.

In most nonlinear systems, superposition theorem no longer can be applied, but benefiting from modulation and demodulation realized through multiplication, the primary mode and the interference mode can be considered separately. Furthermore, the reference signal for demodulation, *cos*(*ω_d_t* + *φ_od_*), can be decomposed into *cosφ_od_* · *cos*(*ω_d_t*) − sin*φ_od_* · *sin*(*ω_d_t*). For clarity, the reference signal in Figure 5 is replaced with *cos*(*ω_d_t*) and the two modes in the sense mode are unified as:

(7) $$G\left(s\right)=\frac{1/m}{s^{2}+(\omega /Q)\cdot s+\omega^{2}}$$

Applying Laplace Transform to Equation (6) after substituting $\cos\left(\omega_{d}t\right)=\frac{1}{2}\left(e^{j\omega_{d}t}+e^{-j\omega_{d}t}\right)$ into it yields

(8) $$F_{c}\left(s\right)=-2m_{c}A_{x}\omega_{d}\frac{1}{2}\left[\Omega \left(s+j\omega_{d}\right)+\Omega \left(s-j\omega_{d}\right)\right]$$

where *Ω*(*s*) is the Laplace Transform of *Ω*(*t*). Similarly, the Laplace Transform of *c*(*t*) can be obtained as

(9) $$C\left(s\right)=\frac{1}{2}K_{\text{pre}}Y\left(s+j\omega_{d}\right)+Y(s-j\omega_{d})$$

Substituting Equation (8) into *Y*(*s*) = *F_c_*(*s*)*G*(*s*) and rearranging the obtained equation together with Equation (9) yield

(10) $$C\left(s\right)=-2m_{c}A_{x}\omega_{d}K_{\text{pre}}\frac{1}{4}\left\{\left[G\left(s+j\omega_{d}\right)+G\left(s-j\omega_{d}\right)\right]\Omega \left(s\right)+G\left(s-j\omega_{d}\right)\Omega \left(s-2j\omega_{d}\right)+G\left(s+j\omega_{d}\right)\Omega \left(s+2j\omega_{d}\right)\right\}$$

The last two terms in Equation (10) are high harmonics with respect to *Ω*(*s*) and will be negligible after the LPF. A conceptual ideal LPF is introduced here. Hence, the Laplace Transform of the output of the gyroscope can be written as

(11) $$R\left(s\right)=-2m_{c}A_{x}\omega_{d}K_{\text{pre}}\cdot \frac{1}{4}\left[G\left(s+j\omega_{d}\right)+G\left(s-j\omega_{d}\right)\right]\Omega \left(s\right)$$

Denote *T*(*s*) = [*G*(*s* + *jω_d_*) + *G*(*s* − *jω_d_*]/4. Substituting Equation (7) into it gives

(12) $$T\left(s\right)=\frac{\left(s^{2}+\omega /Q\cdot s+\omega^{2}-\omega_{d}^{2}\right)/2m}{\left(s^{2}+\omega /Q\cdot s+\omega^{2}+\omega_{d}^{2}+2\omega \omega_{d}\sqrt{1-1/4Q^{2}}\right)\left(s^{2}+\omega /Q\cdot s+\omega^{2}+\omega_{d}^{2}-2\omega \omega_{d}\sqrt{1-1/4Q^{2\;}}\right)}$$

In a similar way, when the reference signal for demodulation is replaced with *sin*(*ω_d_ t*) in Figure 5, it can be derived that

(13) $$T\left(s\right)=\frac{\left(\omega_{d}s+\frac{1}{2}\omega \omega_{d}/Q\right)/m}{\left(s^{2}+\omega /Q\cdot s+\omega^{2}+\omega_{d}^{2}+2\omega \omega_{d}\sqrt{1-1/4Q^{2}}\right)\left(s^{2}+\omega /Q\cdot s+\omega^{2}+\omega_{d}^{2}-2\omega \omega_{d}\sqrt{1-1/4Q^{2}}\right)}$$

So, when the ODP of the reference signal is considered, by combining Equations (12) and (13), we can obtain that

(14) $$T\left(s\right)=\frac{1}{m}\frac{\frac{1}{2}\cos\varphi_{od}\cdot \left(s^{2}+\omega /Q\cdot s+\omega^{2}-\omega_{d}^{2}\right)-\sin\varphi_{od}\cdot \left(\omega_{d}s+\frac{1}{2}\omega \omega_{d}/Q\right)}{\left(s^{2}+\omega /Q\cdot s+\omega^{2}+\omega_{d}^{2}+2\omega \omega_{d}\sqrt{1-1/4Q^{2}}\right)\left(s^{2}+\omega /Q\cdot s+\omega^{2}+\omega_{d}^{2}-2\omega \omega_{d}\sqrt{1-1/4Q^{2}}\right)}$$

For relatively low frequencies and by using the Taylor series, Equation (14) can be simplified as

(15) $$T\left(s\right)\approx \frac{1}{m}\frac{\frac{1}{2}\cos\varphi_{od}\cdot \left(\omega^{2}-\omega_{d}^{2}\right)-\sin\varphi_{od}\cdot \left(\omega_{d}s+\frac{1}{2}\omega \omega_{d}/Q\right)}{{\left(\omega +\omega_{d}\right)}^{2}\left[s^{2}+\omega /Q\cdot s+{\left(\omega -\omega_{d}\right)}^{2}+\omega \omega_{d}/4Q^{2}\right]}$$

Then taking the primary mode and the interference mode into account, the complete form of *T*(*s*) will be modified as

(16) $$\begin{array}{l} T\left(s\right)\approx \frac{\left[\frac{1}{2}\cos\varphi_{od}\cdot \left(\omega_{ny1}^{2}-\omega_{d}^{2}\right)-\sin\varphi_{od}\cdot \omega_{d}\left(s+\frac{1}{2}\omega_{ny1}/Q_{1}\right)\right]/m_{y}}{{\left(\omega_{ny1}+\omega_{d}\right)}^{2}\left[s^{2}+\frac{\omega_{ny1}}{Q_{1}}\cdot s+{\left(\omega_{ny1}-\omega_{d}\right)}^{2}+\frac{\omega_{ny1}\omega_{d}}{4Q_{1}^{2}}\right]}\\ +\frac{\alpha \left[\frac{1}{2}\cos\varphi_{od}\cdot \left(\omega_{ny2}^{2}-\omega_{d}^{2}\right)-\sin\varphi_{od}\cdot \omega_{d}\left(s+\frac{1}{2}\omega_{ny2}/Q_{2}\right)\right]/m_{y}}{{\left(\omega_{ny2}+\omega_{d}\right)}^{2}\left[s^{2}+\frac{\omega_{ny2}}{Q_{2}}\cdot s+{\left(\omega_{ny2}-\omega_{d}\right)}^{2}+\frac{\omega_{ny2}\omega_{d}}{4Q_{2}^{2}}\right]} \end{array}$$

The overall transfer function of the sensor output can be obtained as

(17) $$H\left(s\right)≜\frac{R\left(s\right)}{\Omega \left(s\right)}=-2m_{c}A_{x}\omega_{d}K_{\text{pre}}T\left(s\right)$$

where *T* (*s*) is described in Equation (16).

### 4.2. The Impact of the Interference Mode on the Frequency Response and the Bandwidth of Gyroscope

**Figure 6.**
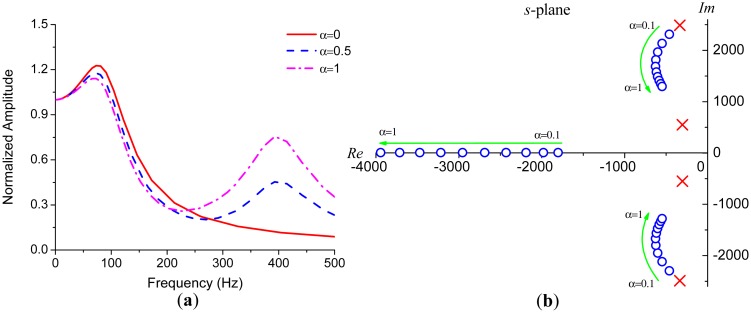
(**a**) The impact of the interference mode on the frequency response; (**b**) The movements of zeros and poles in *s*-plane as *α* gets larger.

Considering the complexity of the fourth-order system described in Equation (16), numerical simulations are more straightforward than analytical methods in evaluating the frequency response of the gyroscope. Figure 6a demonstrates several frequency responses with different α. The parameters used in these simulations are the same with those in Figure 4a except *Q*_1_ = *Q*_2_ = 30. The primary resonance peak is weakened by the existence of the interference mode. This effect derives from the fact that the primary pair of zeros of Equation (16) gets closer to the primary poles as *α* gets larger, as illustrated in Figure 6b where the blue circles indicate the zeros and the red crosses indicate the poles.

**Figure 7.**
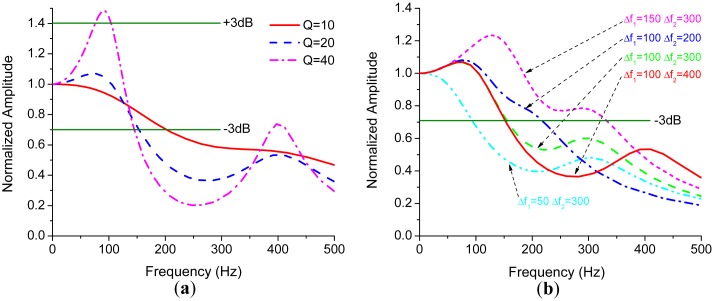
(**a**) The frequency responses with different *Q*; (**b**) The frequency responses with variations of frequency differences.

Figure 7a shows that *Q*-factor drastically affects the resonance peaks. The bandwidth of the gyroscope will be limited by the first peak if it exceeds +3 dB point for some high-*Q* gyroscopes, especially for vacuum packaged ones. However, for the gyroscopes operating at atmospheric pressure, the bandwidth is often limited by −3 dB point.

Besides *Q*-factor, the bandwidth is directly related to the difference between the natural frequency of sense mode and that of drive mode. We denote Δ*ω*_1_ = *ω_ny_*_1_ − *ω_d_*, Δ*ω*_2_ = *ω_ny_*_2_ − *ω_d_* for convenience. Figure 7b is obtained by varying Δ*ω*_1_ and Δ*ω*_2_, while respectively fixing *α* and *Q* as 0.6 and 20. As shown in Figure 7b, the bandwidth significantly decreases as Δ*ω*_1_ decreases and gradually increases as the interference mode approaches to the drive mode. Since the interference mode will not affect the bandwidth drastically, if it is far away from the drive mode, which can be easily observed both in Figures 6a and 7b. The bandwidth can be estimated through the first term in Equation (16) which is a second-order system with an effective *Q*-factor of

(18) $$Q_{e}=\frac{\sqrt{\Delta \omega_{1}^{2}+\omega_{ny1}\omega_{d}/4Q_{1}^{2}}}{\omega_{ny1}}Q_{1}$$

From Equation (18), the flatness of the frequency response can be fast evaluated. The primary resonance peak will appear if $Q_{e}>1/\sqrt{2}$, or equivalently,

(19) $$Q_{1}>\frac{\sqrt{\omega_{ny1}\left(2\omega_{ny1}-\omega_{d}\right)}}{2\left|\Delta \omega_{1}\right|}\approx \frac{\omega_{ny1}}{2\left|\Delta \omega_{1}\right|}$$

As mentioned before, the interference mode weakens the peak, so the actual critical *Q*-factor will be slightly larger than Equation (19) predicts.

It should be noted that the amplitude responses in Figure 7 are normalized with respect to zero frequency points. Revealed by Equation (17), the frequency difference also affects the amplitude of the zero frequency point which reflects the scale-factor of the micro-gyroscope. This will be discussed in the next section.

## 5. Scale-Factor of the Micro-Gyroscope

**Figure 8.**
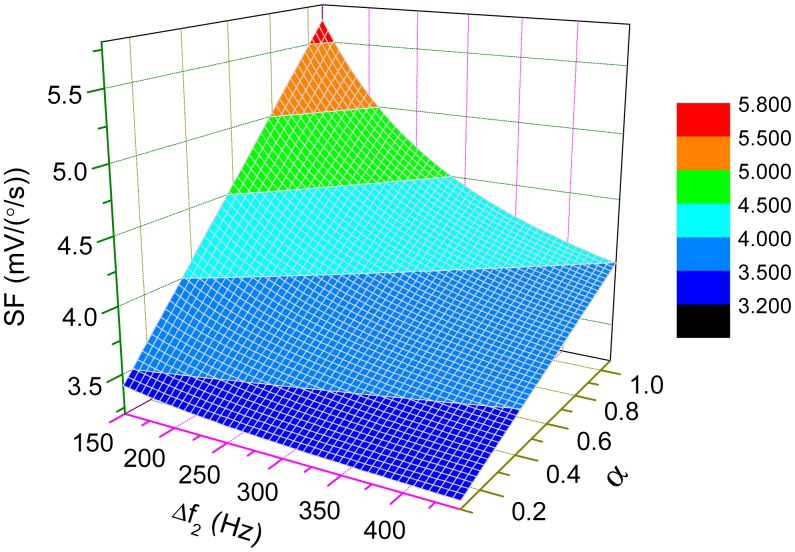
The change of scale-factor with variations of Δ*ω*_2_ and *α*.

The scale-factor of the micro-gyroscope is defined as the ratio of the sensor output to the angular rate applied. Letting *s* = *j*0 in Equation (17) gives the scale-factor of the gyroscope

(20) $$SF=K\left[\frac{\cos\varphi \cdot \left(\omega_{ny1}^{2}-\omega_{d}^{2}\right)-\sin\varphi \cdot \left(\omega_{ny1}\omega_{d}/Q_{1}\right)}{{\left(\omega_{ny1}+\omega_{d}\right)}^{2}\left(\Delta \omega_{1}^{2}+\frac{\omega_{ny1}\omega_{d}}{4Q_{1}^{2}}\right)}+\alpha \frac{\cos\varphi \cdot \left(\omega_{ny2}^{2}-\omega_{d}^{2}\right)-\sin\varphi \cdot \left(\omega_{ny2}\omega_{d}/Q_{2}\right)}{{\left(\omega_{ny2}+\omega_{d}\right)}^{2}\left(\Delta \omega_{2}^{2}+\frac{\omega_{ny2}\omega_{d}}{4Q_{2}^{2}}\right)}\right]$$

where *K* = − (*m_c_*/ *m_y_*) *A_x_ω_d_K_pre_* and *φ* = *φ_od_* · The impact of the interference mode on the scale-factor is simulated in Figure 8. The parameters are *m_c_*/*m*y ≈ 1, *A_x_* = 1μm *f_d_* = 2800 Hz, *f_ny_*_1_ = 2900 Hz, *K_pre_* = 5V/μm and *Q*_1_ = *Q*_2_ =20. Either the interference mode getting stronger or locating closer to the drive mode can increase the scale-factor. However, as mentioned before, the interference mode arises from imperfections in structures which are uncontrollable during the manufacturing process. The amplitude of the interference mode varies from gyroscope to gyroscope and is sensitive to temperature change. Thus, the interference mode should be designed as far away from the working modes as possible to guarantee the stability of the scale-factor.

**Figure 9.**
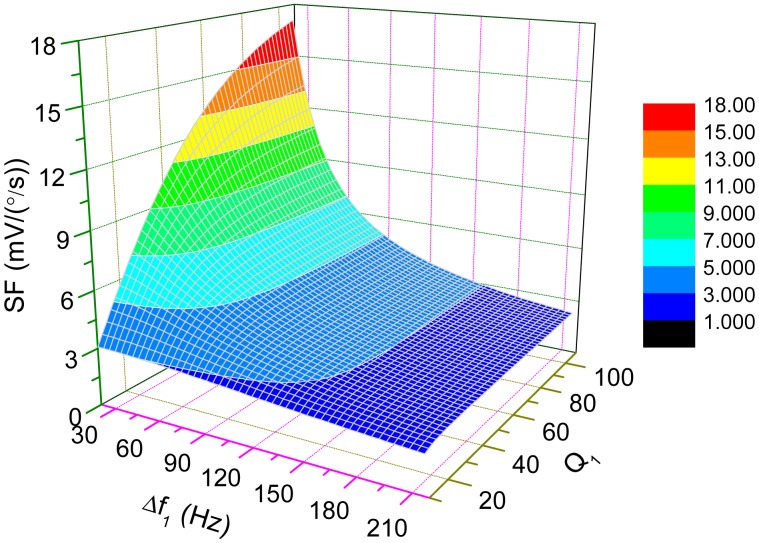
The change of scale-factor with the variations of Δ*ω*_1_ and *Q*_1_.

The change of scale-factor with the variations of Δ*ω*_1_ and *Q* is demonstrated in Figure 9 where *f_ny_*_2_ and *α* are set as 3200 Hz and 0.6, respectively. For high-*Q* cases, the scale-factor is nearly inversely proportional to Δ*ω*_1_, which implies that the sensitivity of the micro-gyroscope will become very large if Δ*ω*_1_ is small. When Δ*ω*_1_=0, which is known as mode-matched condition, the ODP will be close to −90°. Then, the scale-factor can be approximately simplified as

(21) $$SF\approx K\left[\frac{Q_{1}}{\omega_{d}^{2}}+\alpha \frac{\omega_{ny2}\omega_{d}/Q_{2}}{{\left(\omega_{ny2}+\omega_{d}\right)}^{2}\Delta \omega_{2}^{2}}\right]$$

**Figure 10.**
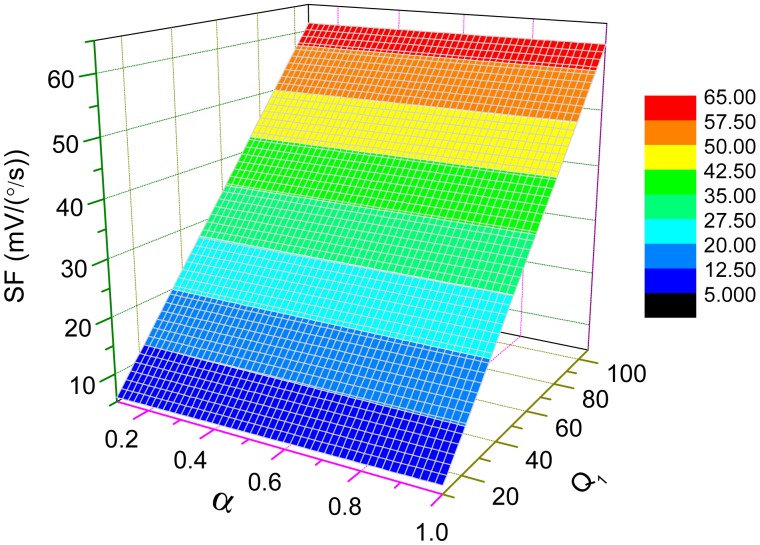
The dependence of scale-factor on *Q*-factor and *α* under mode-matched condition.

Figure 10 illustrates the dependence of the scale-factor on *Q*-factor and *α* under this mode-matched condition. From the plot in Figure 10, the scale-factor is nearly proportional to *Q* which may achieve more than thousands in vacuum packaged micro-gyroscopes. The sensitivity of those micro-gyroscopes can be greatly improved under the mode-matched condition.

Interestingly, back to Figure 9 for low-*Q* cases, the scale-factor will not increase drastically as Δ*ω*_1_ approaches to zero. Even under mode-matched condition, the scale-factor is limited by low *Q*-factor. As mentioned in the last section, the open-loop bandwidth of the micro-gyroscope is dominantly determined by Δ*ω*_1_. So, the improvement of the sensitivity by decreasing the bandwidth is no longer obvious.

**Figure 11.**
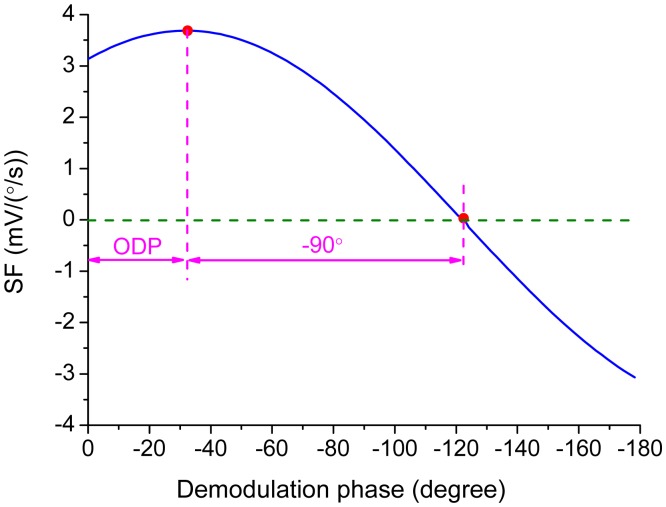
The relationship between the scale-factor and the demodulation phase.

In practice, the demodulation phase can hardly be exactly the same with the ODP. The variation of the scale-factor is shown in Figure 11 when the error of the demodulation phase, *φ* − *φ_od_*, is introduced by varying *φ* from 0° to −180° in Equation (20). The scale-factor reaches the maximum value when the demodulation phase is set as the ODP determined by Equation (4) while the scale-factor becomes zero when the demodulation phase is delayed another 90°. This implies that the reference signal is orthogonal to the Coriolis response. The slope of the curve in Figure 11 at the ODP point is zero while the slope becomes the steepest at the point where the scale-factor reaches zero. This relationship can be used in experiments to find out the exact ODP.

## 6. Results

**Figure 12.**
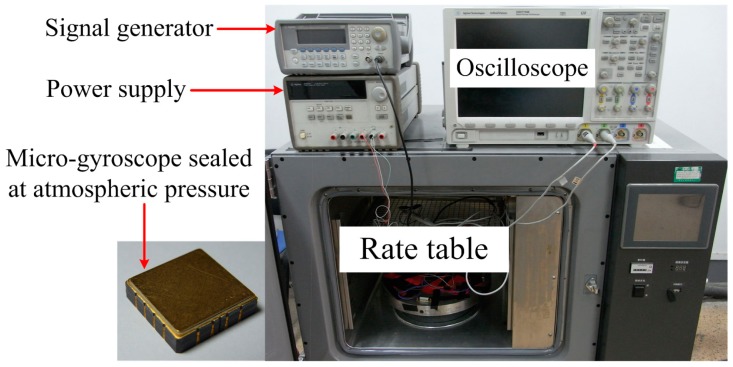
The photos of the micro-gyroscope and the experiment setups.

**Figure 13.**
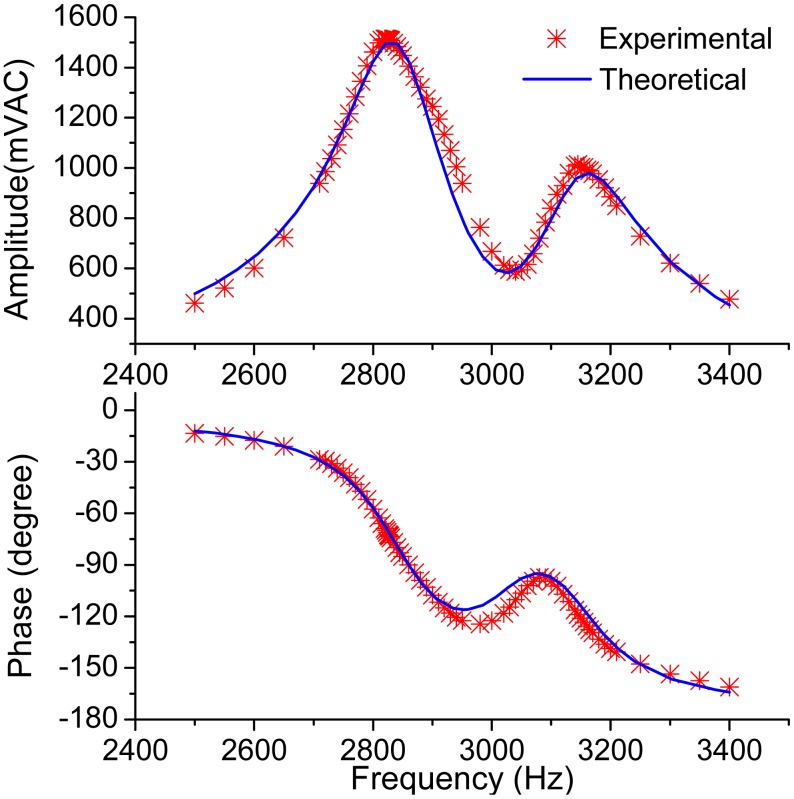
The measured frequency response and the theoretical response of the sense mode.

To verify the theoretical analyses above, a micro-gyroscope designed by our research team, shown in Figure 12 together with the photo of the experiment setups, was tested at atmospheric pressure. Half of the sense combs were used as force feedback combs to excite the sense mode. Figure 13 shows the measured frequency response of the sense mode, along with the theoretical response calculated from Equation (2) with the experimentally determined parameters. In order to fit the data, the total gain of Equation (2) was adjusted as 2.8 × 10^10^.

**Figure 14.**
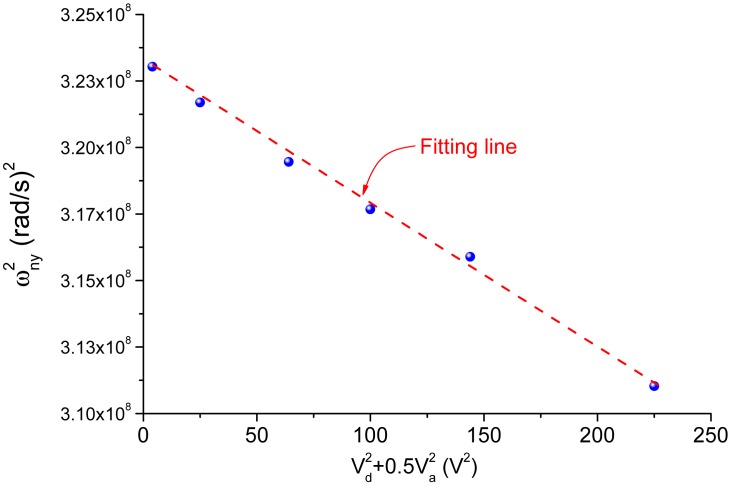
The negative electrostatic stiffness of the sense mode.

As mentioned before, the sense combs are configured as squeeze mode which has an effect of negative electrostatic stiffness. When a voltage is applied to the electrodes, the stiffness of the sense mode will decrease in accordance with the following equation

(22) $$\omega_{ny}=\sqrt{\omega_{ny0}^{2}-\kappa \left(V_{d}^{2}+V_{a}^{2}/2\right)}$$

where *ω_ny_*_0_ is the initial natural frequency of the sense mode; κ is a constant coefficient related to the details of the structure; *V_d_* is DC voltage while *V_a_* is the amplitude of AC voltage. This effect derives from the nonlinear property of the squeeze combs. Thus, the data plotted in Figure 13 did not reflect the real parameters of the sense mode because in order to obtain the data, an AC voltage biased by a DC voltage must be applied to excite the sense mode. To obtain the parameters of the sense mode without the influence of test voltages, the relationship between the natural frequency and the test voltages was measured, as shown in Figure 14 where $\omega_{ny}^{2}$ is plotted as a linear function of $V_{d}^{2}+V_{a}^{2}/2$. From Equation (22), the absolute value of the slope of the line is the coefficient *κ* and the vertical intercept is the square of the initial natural frequency. By utilizing the linear fitting method, the coefficient *κ* and the initial natural frequency were obtained. The modified parameters of the sense mode are listed in Table 1. From the mode locations demonstrated in Figure 2, the observed interference mode is believed to be the anti-phase torsional mode.

**Figure 15.**
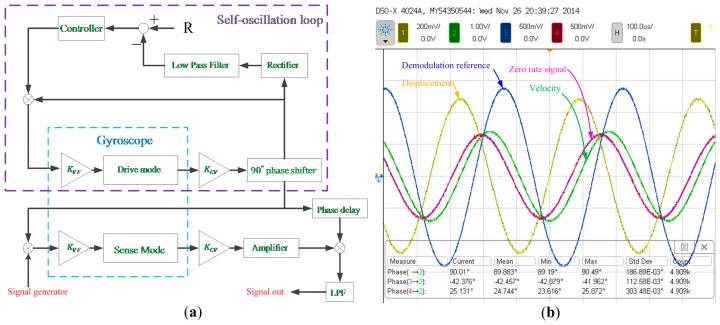
(**a**) The block diagram of the circuits for the bandwidth experiment; (**b**) The phase relationships of the signals in the system when no angular rate was applied.

The frequency response of the micro-gyroscope was measured by using virtual rate table method which is described in [21] in detail. Figure 15a illustrates the block diagram of the control and sense circuits for the experiment. The phase delay block, which was implemented with an all-pass filter, was set as about −42.5° while the theoretical ODP is calculated as −43.7° by using the parameters listed in Table 1.

The quadrature response would have a phase delay of 43.7° with the quadrature force which is always in-phase with the displacement signal. From Figure 15b, it can be seen that the zero-rate signal had a phase delay of 65° with the displacement signal. In other words, the zero-rate signal contained not only the quadrature response but also the response coming from the coupling damping force, which was in-phase with the velocity signal. Through the phase relations mentioned above, the magnitude ratio of the quadrature force to the coupling damping force can be determined as 2.56 by using simple trigonometric identities. The ODP cannot be obtained by the phase difference between the zero-rate signal and the displacement signal because the mentioned magnitude ratio remains unknown unless the ODP is already obtained.

**Figure 16.**
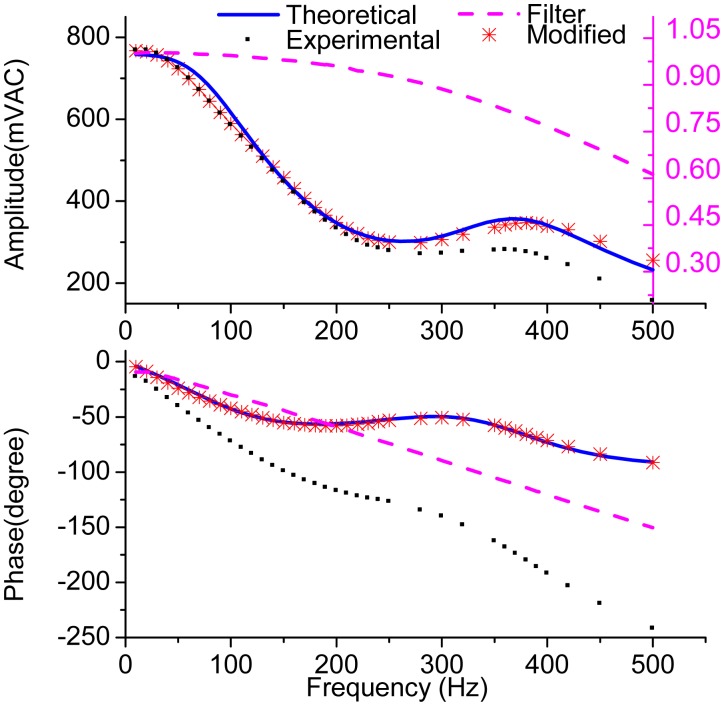
The measured frequency response and the theoretical frequency response of the micro-gyroscope.

Figure 16 shows the measured frequency response of micro-gyroscope and the theoretical response calculated from Equation (16) by using the parameters listed in Table 1. In order to filter the harmonics introduced by the demodulation, the LPF at the output stage is necessary in the experiment. However, in the theoretical analysis, an ideal LPF is introduced. Considering the influence of the LPF, the amplitude attenuation and phase delay introduced by the LPF were also measured and are plotted with the pink dash line in Figure 16. Then, the measured amplitude response of the gyroscope was divided by the attenuation factor and the measured phase response was added with the phase delay. The modified data are plotted with red stars in Figure 16. In addition, to plot the theoretical curve, the negative electrostatic stiffness was considered and the total gain was adjusted as 3.4 × 10^10^ to fit the experimental data.

**Figure 17.**
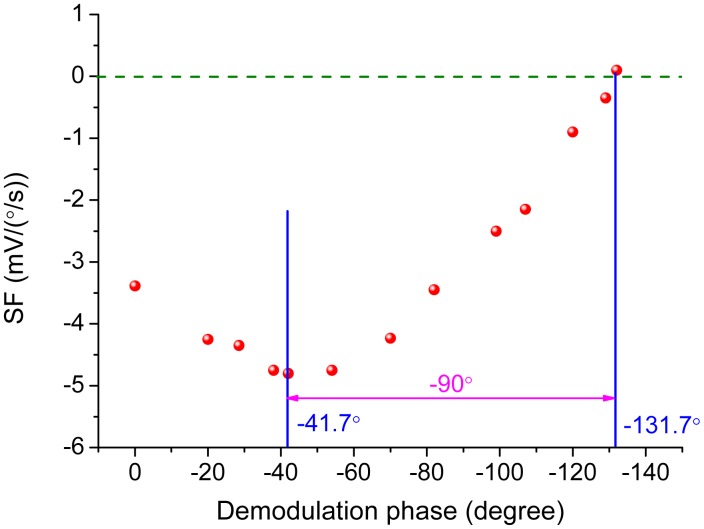
The measured scale-factor with different demodulation phases.

The change of the scale-factor with the variation of demodulation phase was measured by real rate table and is demonstrated in Figure 17. The demodulation phase was adjusted by the RC parameters in the all-pass filter. The scale-factor achieved the maximum value as −4.8 mV/(°/s) at the demodulation phase of −41.7° and decreased to zero at the demodulation phase of −131.7°. The ODP was obtained as −41.7° from Figure 17 and was in close agreement with the theoretical value calculated as −43.7°.

## 7. Conclusions

The cost of micro-gyroscope is still too high for certain applications. Developing high-performance micro-gyroscopes operating at atmospheric pressure is one of the effective ways to further lower the cost. In this paper, the frequency response and scale-factor of the gyroscope with low *Q*-factor are studied in detail.

In order to extract the Coriolis response completely, a demodulation phase should be introduced in the sense circuits. The ODP is obtained through the transfer function of the sense mode which consists of the primary mode and the interference mode. The ODP will decrease as the amplitude of the interference mode or the *Q*-factor increases. The approximate transfer function from the angular rate input to the gyroscope output is presented in consideration of the interference mode and the ODP. Through numerical simulations, it can be concluded that the flatness of the frequency response is mainly determined by *Q*-factor of the sense mode, and the appearance of the interference mode will decrease the peak value of the amplitude response if there is a peak. The impact of the interference mode on the frequency response will get stronger as the frequency difference between the interference mode and the drive mode gets smaller. Although the scale-factor becomes larger when the interference mode becomes larger or gets closer, the increase of the sensitivity is unstable and should be avoided as much as possible. For micro-gyroscopes with low *Q*-factor, the mechanical sensitivity is limited by *Q*-factor even under mode-matched condition. The improvement of the sensitivity by reducing the frequency difference between the working modes is not so significant compared with the vacuum packaged gyroscopes.

It is shown that the theoretical analyses are in close agreement with the results of the experiments. The deduced transfer functions and the simulations carried out can provide constructive guidance on bandwidth and sensitivity designs of the micro-gyroscopes operating at atmospheric pressure.

## Figures and Tables

**Table 1.** The parameters of the sense mode and the driving frequency from the experiments.

**Parameters**	**Unit**	**Before modification**	**After modification**
*f_d_*	Hz	2785.6	unchanged
*f_ny_*_1_	Hz	2844	2862
*f_ny_*_1_	Hz	3145	3159
*Q*_1_	-	16	unchanged
*Q*_2_	-	17	unchanged
a	-	0.64	unchanged
